# How subjective well-being, patient-reported clinical improvement (PROMs) and experience of care (PREMs) relate in an acute psychiatric care setting?

**DOI:** 10.1192/j.eurpsy.2023.12

**Published:** 2023-02-17

**Authors:** Elisabetta Scanferla, Katherine de Bienassis, Bernard Pachoud, Philip Gorwood

**Affiliations:** 1 CMME, GHU Paris Psychiatrie et Neurosciences, Hôpital Sainte-Anne, Paris, France; 2 Université Paris Cité, ED 450, Paris, France; 3 Organisation for Economic Co-operation and Development, Paris, France; 4 Université Paris Cité, INSERM, U1266 (Institute of Psychiatry and Neuroscience of Paris), Paris, France

**Keywords:** patient-reported experience measures, patient-reported outcome measures, psychiatry, mental health care, quality indicators

## Abstract

**Background:**

Patient-reported outcome measures (PROMs) and patient-reported experience measures (PREMs) are increasingly acknowledged as critical tools for enhancing patient-centred, value-based care. However, research is lacking on the impact of using standardized patient-reported indicators in acute psychiatric care. The aim of this study was to explore whether subjective well-being indicators (generic PROMs) are relevant for evaluating the quality of hospital care, distinct from measures of symptom improvement (disease-specific PROMs) and from PREMs.

**Methods:**

Two hundred and forty-eight inpatients admitted to a psychiatric university hospital were included in the study between January and June 2021. Subjective well-being was assessed using standardized generic PROMs on well-being, symptom improvement was assessed using standardized disease-specific PROMs, and experience of care using PREMs. PROMs were completed at admission and discharge, PREMs were completed at discharge. Clinicians rated their experience of providing treatment using adapted PREMs items.

**Results:**

Change in subjective well-being (PROMs) at discharge was significantly (*p* < 0.001), but moderately (*r* ^2^ = 28.5%), correlated to improvement in symptom outcomes, and weakly correlated to experience of care (PREMs) (*r* ^2^ = 11.0%), the latter being weakly explained by symptom changes (*r* ^2^ = 6.9%). Patients and clinicians assessed the experience of care differently.

**Conclusions:**

This study supports the case for routinely measuring patients’ subjective well-being to better capture the unmet needs of patients undergoing psychiatric hospital treatment, and the use of standardized patient-reported measures as key indicators of high quality of care across mental health services.

## 1. Introduction

Two overlapping but distinct patient care approaches have gained political and professional momentum over the past decades: patient-centred care (PCC), defined as “providing care that is respectful of and responsive to individual patient preferences, needs and values, and ensuring that patients’ values guide all clinical decisions” [1], and value-based healthcare (VBHC) where patient-centred outcome measures determine the evaluation of the efficacy of care [2, 3]. Both approaches encourage health systems to reframe provided services so they are person-oriented [4] and reflect patients’ preferences [5, 6]. Newly tailored measures have emerged to capture and quantify the patient’s voice. These metrics are complementary to clinician-reported measures, which have a limited ability to capture key aspects of patient needs and outcomes [7–10]. Best practices in collecting patient-reported data involve the use of standardized, validated, self-administered questionnaires, to capture patient-reported outcome measures (PROMs). PROMs can be either (1) “generic” (non “disease-specific”), which measure various aspects of quality of life, or (2) “disease-specific,” that is concentrating on the perception of symptoms and health status of a particular group of patients or conditions [11]. Patient-reported metrics also include patient-reported experience measures (PREMs), which are designed to discern patients’ perception of their experience with health services and healthcare delivery [12]. In this article, we refer to “patient-reported measures” as comprising both PROMs and PREMs.

Researchers, policy makers, and patient groups have documented numerous benefits of utilizing patient-reported measures across medical specialties [6, 13–17]. In clinical management, the use of patient-reported metrics has been found to correspond with increases in clinician awareness and consideration of patient needs, values, and preferences [4], and can be used to support shared decision-making during treatment [7, 18–21]. Embedding harmonized patient-reported measures in routine care can lead to their more consistent use, increase systematic uptake for measuring care quality, and generate information to inform the implementation of health policies [22].

In mental health research and practice, patient-reported measures have gained growing attention. They are being recognized as an effective instrument for improving high-quality care [7, 20, 23] and performance across its three core dimensions: effectiveness, safety, and patient-centredness [24]. For example, the Organisation for Economic Co-operation and Development (OECD) is promoting the routine use of patient-reported measures to facilitate national and international comparisons of these measures, and in policy making [25]. OECD is testing in particular standardized indicators in the field of mental health care, and in particular PROMs of subjective well-being [25]. Subjective well-being dimension encompasses elements of good psychological functioning, notably affective reactions of individuals to their experiences [22] (presence of positive and negative feelings) [26], *eudaimonia,* the condition of human flourishing [27], and life evaluation (satisfaction and worthwhileness). Subjective well-being is a distinct concept from health-related quality of life (HRQoL), a multidimensional construct that consists of three broad domains—physical, psychological, and social functioning—and refers in particular to individuals’ cognitive assessment of the impact of their health on their daily lives [28]. Thus, it is important to capture subjective well-being in addition to traditional HRQoL data.

Improving well-being is particularly relevant for individuals experiencing severe mental disorders where the chronicity of particular symptoms can adversely affect their well-being and general quality of life [16, 29]. Nevertheless, limited research has investigated the use of standardized patient-reported indicators in this patient population [30], and their potential to fill the gap between clinician and patient views on well-being [31]. Recent research includes Shadmi and colleagues [32] whose findings demonstrate that psychiatric service users’ reports of their quality of life may predict the risk of future hospitalization. In addition, a research study conducted by Mendlovic et al. [33] analysed data collected from people recently admitted to a psychiatric hospital, finding a strong correlation between patients’ assessment of patient-rated HRQoL, experience of care, and overall severity of their disease. However, none of these studies specifically addressed the area of subjective well-being.

The main objective of this study was to evaluate the relevance of patient-reported measures to capture the domain of subjective well-being as an indicator of the quality of hospital care, distinct from, though complementary to, measures of symptom improvement and patient experience reported by patients.

We hypothesized that the patients’ measures of subjective well-being (generic PROMs) at discharge may only be partially related to the satisfaction with the experience of care (PREMs) and symptom improvement (disease-specific PROMs), and potentially unrelated to clinician experience of provided care.

## 2. Methods

### Participants

This was a prospective cross-sectional study that assessed data collected in routine care of patients admitted to a university group psychiatric hospital. Consecutively hospitalized patients in two departments of the *GHU Paris psychiatrie et neurosciences*, a university hospital group, between 31 January 2021 and 30 June 2021, were assessed for eligibility (*N* = 379). The first participating department was a university department specialized in mood and eating disorders; the second was a general psychiatric department providing care to the residents of a given geographic area suffering from diverse severe psychiatric disorders. Inclusion criteria were age > 18 years, hospitalization of two weeks or more, with a principal diagnosis code of mental health and behavioral disorders (ICD-10 codes F10-F69 and F90–99). The inclusion criteria related to the length of hospitalization is due to the construct of the World health organization well-being index (WHO-5) assessment tool, which covers a two-week look-back period.

**Figure 1.**
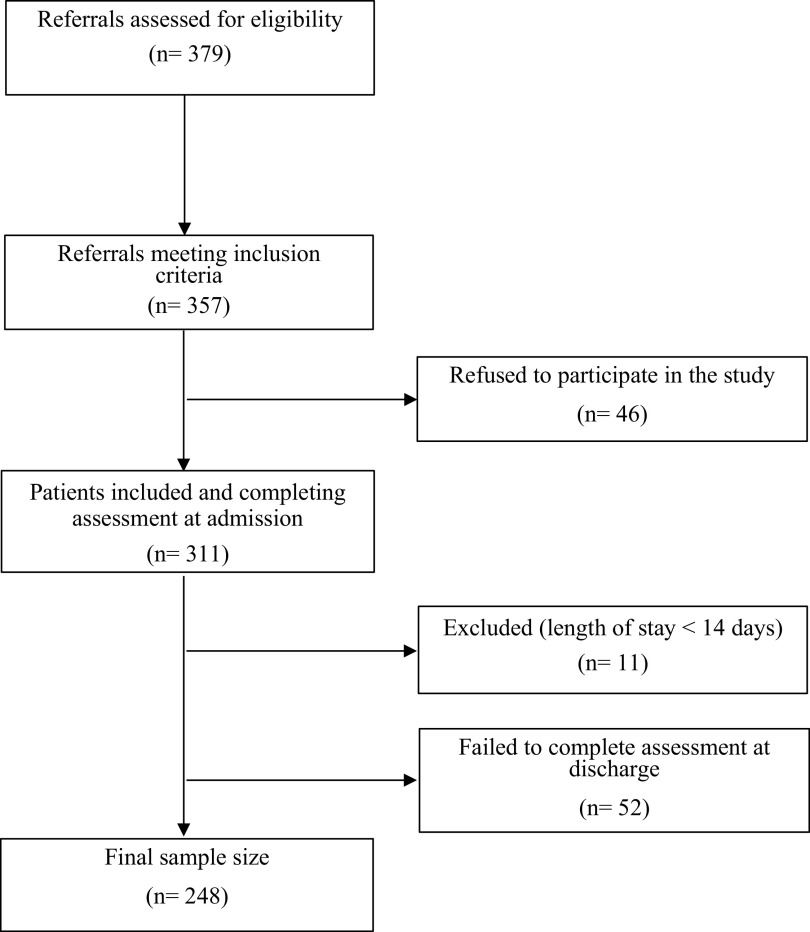
Flow chart of participants’ selection.

Of the 357 inpatients eligible for inclusion, 311 were enrolled in the study, 52 did not complete the assessment at discharge, and 11 were excluded because the final length of their stay did not meet the inclusion criteria (see Figure 1). A total of 248 participants were included in the final sample (dropout rate, 20.2%). Seventy-six (30.6%) of the participants suffered from eating disorders, 72 (29.0%) from psychotic disorders, 46 (18.5%) from mood disorders, 44 (17.8%), were hospitalized for suicidal crises and 10 (4.0%) suffered from alcohol-use disorder (AUD). The mean age of enrolment was 37.0 years(SD = 14.1, range: 18–85); 74.2% (*n* = 184) were female. The mean length of hospitalization was 45.6 days (SD = 32.5, range: 14–222) (see Table 1). Patients suffering from psychotic disorders and AUD were not asked to complete the disease-specific PROMs to align with existing departmental workflows. Consequently, all statistics are based on *N* values of 248 or 166, respectively. Participants were assessed using the Mini International Neuropsychiatric Interview (M.I.N.I) [34], a brief structured diagnostic interview evaluating the most common psychiatric disorders. Evaluations were conducted by trained clinicians. This information was used to inform the principal diagnosis. Non-French-speaking patients and those having a primary neurological disorder were excluded from assessment with the M.I.N.I.

**Table 1.** Main demographic and clinical characteristics of the population sample (*N* = 248).

	*N*	Mean	SD	Range	%
Variable (continuous)					
Age, years	248	37.0	14.1	18–85	
Length of hospitalization	248	45.6	32.5	14–222	
Variable (categorical)					
Gender					
Female	184				74.2
Male	64				25.8
Diagnosis					
Eating disorders	76				30.6
Psychotic disorders	72				29.0
Mood disorders	46				18.5
Suicidal crisis	44				17.8
Alcohol-use disorders	10				4.0

Abbreviation: SD, standard deviation.

### Clinical assessment

Patients were invited to complete the questionnaires assessing subjective well-being (generic PROMs) and changes in symptoms that caused the hospitalization (disease-specific PROMs), at day of admission (+ maximum of 48 h) and at day of discharge (+ maximum of 48 h). At discharge, they also completed a questionnaire assessing their experience of care (PREMs). PREMs and PROMs collection was conducted via paper-and-pencil questionnaires. The patients’ primary clinicians completed a self-administered questionnaire about their experience of caregiving. Patients’ sociodemographical data (age, sex) were collected from the participants’ medical records.

### Instruments

#### Generic PROMs

The selected instruments followed the survey of the OECD PaRIS on Mental Health care, 2021 [22] and included:

(a) The World Health Organization Well-Being Index (WHO-5) [35]: a self-report questionnaire consisting of five items that cover important aspects of subjective well-being (cheerfulness, calmness, activity, rest, and interest). The scoring is on a five-point scale, ranging from “All the time” to “At no time” (see Appendix 1). The total score ranges from 0 to 25, with high scores indicating an increased sense of well-being. The WHO-5 has demonstrated adequate psychometric properties across different somatic settings [35–37], with reported Cronbach α coefficients estimated between 0.83 [38] and 0.92 [39]. Studies conducted among adults for monitoring patient response to treatment in psychiatric services, demonstrated the high reliability, validity and clinical utility of the WHO-5 [40].

(b) The OECD Assessment of Subjective Well-being Core Items (two items) module [32] on overall life satisfaction, intended to capture the respondent’s evaluative judgment of how their life is going (item 1), and finding meaning and worth in life (item 2) (see Appendix 1). The total score ranges from 1 to 10, with higher scores indicating an increased satisfaction with life and a higher sense that things in life are worthwhile. The reliability and validity for multiple-item measures of life satisfaction is good, with a Cronbach α of between 0.80 and 0.96 [26].

#### Disease-specific PROMs

(a) *HADS*: The French version of the Hospital Anxiety and Depression Scale (HADS) [41]: a self-assessment scale measuring anxiety (HADS-A) and depression (HADS-D) in general medical populations. For both scales, higher scores indicate more severe psychopathology [41]. The internal consistency for patients of non-psychiatric clinics has been estimated between 0.68 and 0.93 for the HADS-A (mean 0.83), and between 0.67 and 0.90 for the HADS-D (mean 0.82) [42].

(b) *EDI-2*: The Eating Disorder Inventory-2 (EDI-2) [43, 44]: a self-rating inventory with 91 items and 11 subscales designed for the exploration of attitudinal and behavioral dimensions relevant to eating disorders. Higher scores indicate more severe psychopathology [45]. The internal consistency found for the eight original scales of the EDI was above 0.80 for patients suffering from anorexia nervosa, and above 0.60 for healthy subjects [45], while the three added scales showed a Cronbach α ranging from 0.65 (ascetism) to 0.75 (impulse regulation) in an eating disordered sample [46].

(c) *BSS*: Beck Scale for Suicide Ideation [47, 48]: a self-report instrument designed to assess the severity of a patient’s suicidal ideation and to identify a risk of acting on it. The BSS is composed of 21 items that assess: reasons for living or dying, duration and frequency of suicidal thoughts, anticipation of a real attempt, and degree of preparation. Higher values indicate a greater risk of suicide. The Cronbach α coefficient for patients admitted to psychiatric services has been estimated 0.93 and indicated internal consistency [49].

#### PREMs

Patients’ treatment satisfaction was measured using a four-item rating scale adapted from the OECD-Proposed Set of Questions on Patient Experiences with Ambulatory Care [32]. It explores the following dimensions: courtesy and respect; time spent with the clinician; clarity of the explanations and involvement in decisions about care and treatment (see Appendix 1). The scale comprises four responses categories on a 0–3 scale ranging from “Yes, definitely” to “No, definitely not.” Higher scores indicate more positive levels of satisfaction with the experience of care. The internal consistency was greater than 0.80.

#### Clinician-rated experience of care delivered

In order to compare patients’ and clinicians’ experience, clinicians were asked to rate their experience of the treatment delivered to their patients using the PREMs questionnaire adapted to clinicians. The response scale was the same as for the PREMs (see Appendix 2).

### Statistical analysis

All items measured in the study (patient and clinician-reported measures) were standardized to zero mean and unit variance. For comparative purposes, a z-score was used to standardize the results of the disease-specific symptom outcomes questionnaires (PROMs). For continuous measures, the mean and standard deviation (SD) are reported. The relationship between the variables was tested using the Pearson correlation coefficient, Kendal Tau test, and linear regression models. For the generic PROMs, the PREMs, and the CREMs, after verifying their internal consistency (Cronbach α), a synthetic index was calculated by summing the evaluations as performed in previous research and using the same measures [57]. For the generic and disease-specific PROMs, temporal evolution was considered as the difference between the assessment on the day of admission and the assessment on the day of discharge.

A linear regression analysis was performed to test predictors of well-being score changes (standardized difference between admission and discharge scores). The independent variables were symptom change scores (disease-specific PROMs, standardized difference between admission and discharge scores), patient’s experience of care (standardized sum of PREMs scores), and clinicians’ experience of caregiving (standardized sum of CREMs scores).

Finally, a principal component analysis (PCA) was used to identify potential overlapping groups of items of PROMs (well-being and symptoms evolution), PREMs, and CREMs. An orthogonal varimax rotation with Kaiser normalization was used to derive orthogonal factor loadings. Only components with eigenvalues greater than one were considered relevant. Two supplementary qualitative variables (diagnosis and sex) and two quantitative variables (length of hospitalization and age in years) were included in the analysis.

The Ascending Hierarchical Clustering (AHC) algorithm was performed on the PCA-transformed data to identify homogeneous subgroups of patients either suffering from eating and mood disorders, or hospitalized for a suicidal crisis (*N* = 166). For this purpose, the *v*-test [~ *Normal* (0, 1)] was estimated, representing the positive or negative association of the variables with the clusters. The variables with the highest *v*-test values were selected to interpret the main characteristics in each cluster. All tests are two-tailed with a significance level of 5%; in the case of multiple comparisons, Bonferroni adjustment was performed. All statistical analyses were carried out using SPSS, version 26 (IBM Corp. Released 2019. IBM SPSS Statistics for Windows, Version 26.0).

## 3. Results

Subjective well-being as measured with PROMs and PREMs was positively correlated (*r* = 0.331, *p* < 0.0001) across disorders, implying that patients’ satisfaction with the experience of care explained only 11% of the improvement of the well-being scores (*r* ^2^ = 0.109). Subjective well-being measures were negatively correlated (*r* = −0.534, *p* < 0.0001) to symptom severity measures (disease-specific PROMs), indicating that symptom improvement explained 28.5% of the variance in subjective well-being (*r* ^2^ = 0.285). We found a weak, negative, correlation between symptom changes (disease-specific PROMs) and the experience of care (PREMs) (*r* = −0.262, *p* < 0.003), revealing that patients’ satisfaction with the experience of care did not fully explain (6.8%) the improvement in symptoms (*r* ^2^ = 0.068). Moreover, a low level of agreement was found between patients’ and their primary clinicians’ ratings of the hospital care sequence (for all items Kendall test *W* ≤ 0.118, *p* < 0.001). Clinicians rated most items significantly higher than patients (*p* < 0.001).

In addition, linear regression analysis assessing the explanatory value of change in symptoms, patient experience, and clinicians’ experience of care scores (predictor variables) in the participants’ subjective well-being scores (generic PROMs), confirmed that symptom improvement values significantly explained the variability of subjective well-being (*p* < 0.001). The *p*-value observed was 0.078 for PREMs scores (see Table 2).

**Table 2.** Predictors of the variability of subjective well-being: results of linear regression model.

	Well-being (PROMs)^a^		
Independent variables	*Beta*	Student’s *t*	*p*-value (95% CI)
Symptoms change (PROMs)	−0.491	−6.309	<0.000 (−0.613:−0.320)
Patient experience of care (PREMs)^a^	0.140	1.776	0.078 (−0.016:0.296)
Clinician experience of caregiving (CREMs)^a^	0.059	0.769	0.443 (−0.098:0.223)

*Note*: Dependent variable: Change in well-being total score (PROMs). All items of patient and clinician-reported measures were standardized to zero scores. Significant *p*-value ≤ 0.05.

Abbreviations: CI, confidence interval; CREM, clinician-reported experience of delivered care; PREM, patient-reported experience measure; PROM, patient-reported outcome.

a Synthetic index (standardized sum).

Finally, the results of the PCA analysis showed that the patient-reported measures’ items and the clinicians’ experience of caregiving values could be loaded into four relevant factors (eigenvalue >1). This four-factor solution globally explained 60.60% of the overall variance, of which 39.97% related to the first two components. Findings of the orthogonal varimax rotation showed that most of the items were strongly clustered for each of the four factors. The first component “patient-reported outcomes” covered all PROMs (well-being and symptoms improvement). The second, “patient-reported experience,” reflected all the PREM items, and rejected all of the equivalent ratings made by clinicians. The third and fourth factors covered the “clinician-reported experience.” None of these factors overlapped with the “patients-reported experience” factor (see Table 3).

**Table 3.** Principal component analysis of patient-reported outcome measures of well-being and symptoms change, patient-reported experience of care, and clinician-reported experience of delivered care.

	Factor loading			
	Factor 1	Factor 2	Factor 3	Factor 4
	“Patient-reported outcomes”	“Patient-reported experience”	“Clinician-reported experience interaction style”	“Clinician-reported experience attitude”
Eigenvalue	3.03	1.77	1.41	1.06
% of variance explained	25.24	14.73	11.76	8.84
Cumulative % of explained variance	25.24	39.97	51.73	60.57
*Parameters*				
*Well-being (PROMs)*				
Well-being Index	0.755	0.135	0.087	0.039
Meaning and worth	0.737	−0.036	0.084	−0.079
Satisfaction in life	0.696	0.162	−0.076	−0.054
*Symptoms change (PROMs)*				
Symptoms change^a^	−0.739	−0.133	−0.023	0.027
*Patient experience of care (PREMs)*				
Courtesy and respect	−0.012	0.680	0.352	0.077
Time spent with clinician	0.318	0.588	−0.133	0.110
Clarity of explanations	0.108	0.829	0.063	−0.107
Involvement in decisions	0.084	0.806	0.034	−0.054
*Clinician experience of caregiving (CREMs)*				
Courtesy and respect	−0.036	−0.023	0.040	0.878
Time spent with patient	0.117	0.136	0.689	0.301
Clarity of explanations given	−0.106	0.040	0.853	−0.121
Involvement in decisions	0.210	0.026	0.494	−0.410

Abbreviations: CREM, clinician-reported experience of caregiving measure; PREM, patient-reported experience measure; PROM, patient-reported outcome measure.

a Symptoms change: a z-score was used to standardize the results of the disease-specific symptom outcomes questionnaires. The change is the difference between scores at admission and discharge.

Cluster analysis performed with an ascending hierarchical classification model on subjects’ PCA-transformed data to the first three axes identified a total of three homogeneous groups (see Table 4). The distribution of the qualitative variables in the three clusters showed significant differences by gender and diagnosis. The first cluster was characterized by poor well-being and symptom improvement scores (PROMs), significantly low satisfaction with care experience (PREM), and delivered care (CREMs). It consisted mainly of eating disorder patients (67.7% vs 44.9% of the patients suffering from eating disorders in the sample; *p* = 0.003), and, consequently, a high concentration of female patients (96.8% vs 81.9%; *p* = 0.009). The second cluster included patients reporting low increases in PROMs, particularly in symptom outcome scores, a positive experience of care, and a length of hospital stay significantly shorter (28 vs 38 days in the sample). The third cluster was characterized by higher scores of well-being and symptom measures, as well as higher satisfaction with the experience of received care and experience of delivered care. In this cluster, there were significantly more male patients than in the sample (31.9% vs 18.1%; *p* = 0.003).

**Table 4.** Ascending hierarchical clustering: variables with a greater weight according to the *v*-test in each cluster.

	Cluster patients 1			Cluster patients 2			Cluster patients 3		
	*v* test	Mean in clusters (SD)	*p-*value	*v* test	Mean in clusters (SD)	*p-*value	*v* test	Mean in clusters (SD)	*p-*value
*Well-being change (PROMs*)									
Well-being Index	−2.93	−0.46 (0.8)	0.003	*−3.99*	−0.45 (0.8)	<0.001	6.63	0.77 (0.8)	<0.001
Worth in life	−2.83	−0.44 (0.8)	0.005	*−3.68*	−0.41 (0.9)	<0.001	6.74	0.78 (0.8)	<0.001
Satisfaction in life				−5.14	−0.58 (0.8)	<0.001			
*Symptoms change (PROMs*)	3.02	0.47 (0.7)	0.003	4.30	0.48 (0.7)	<0.001	−7.02	−0.81 (0.9)	<0.001
*Patient experience*									
Courtesy	−6.00	−0.94 (11)	<0.001	4.51	0.51 (0.4)	<0.001			
Time spent	−5.39	−0.84 (0.9)	<0.001				3.09	0.36 (0.8)	0.002
Explanations	−6.90	−1.08 (1.1)	<0.001	3.86	0.43 (0.5)	<0.001	2.24	0.26 (0.7)	0.025
Involvement				3.92	0.44 (0.7)	<0.001			
*Clinician experience*				4.51	0.51 (0.4)	<0.001			
Time spent	−2.87	−0.45 (0.9)	0.004	2.02	0.23 (1.0)	0.043			
Explanations	−2.52	−0.507	0.012	2.20	0.25 (0.7)	0.027	3.01	0.35 (0.7)	0.003
Involvement				−2.18	−0.24 (1.1)	0.029	3.01	0.35 (0.7)	0.003

*Note*: Following standardization for all variables, the overall mean = 1 and standard deviation = 0. Significant *p*-value ≤ 0.05.

Abbreviations: CREM, clinician-reported experience of delivered care measure; PREM, patient-reported experience measure; PROM, patient-reported outcome measure.

## 4. Discussion

This study investigated whether the domain of subjective well-being is a relevant indicator of the quality of hospital care, distinct from patient-reported measures of symptom improvement and satisfaction with care. Findings confirmed our hypothesis showing that across mental disorders improvement in subjective well-being was weakly correlated to experience of care and moderately, negatively, correlated to higher prevalence of symptoms. Improvement in symptoms was found to be the strongest predictor of increase in subjective well-being at discharge, but only explained a moderate part of its variance. We also observed that patient-reported measure scores differed between homogenous groups of patients and were potentially driven by their diagnosis: patients suffering from eating disorders were over-represented in the cluster characterized by poorer improvements in symptoms and a less satisfactory care experience.

Overall, the results of the present study suggest that a positive experience of care and the reduction of symptoms, as a traditional goal of acute treatment for psychiatric disorders, only partially contributes to the global sense of well-being at the end of a hospital stay. This provides evidence that the measures of patient-centredness and effectiveness of care (generic PROMs, such as well-being measures, and disease-specific PROMs, such as symptom improvement) are distinct and yet and should both be incorporated separately in clinical practice for comprehensive assessment [50, 51].

These findings are consistent with previous research exploring how patient-reported measures interact, and describing a weak association between experience and outcome measures [52–54]. Black et al.’s [55] findings highlighted that having a positive experience of care can increase symptom improvement scores by approximately 4%, and health-related quality of life scores by approximately 2%. Interestingly, in the clinical field of mental health, Mendlovic et al. [33] reported a stronger correlation coefficient (*r* = 0.57) between patient’s ratings of their quality of life (generic PROMs) and experience of care, whereas our study showed a weak relationship between subjective well-being scores (generic PROMs) and experience of care. This highlights the value of well-being measures: they may contribute deeper insight into what matters to patients, notably in contexts where the hospital treatment fails to achieve full symptom remission [56–58]. For example, by capturing patients’ emotions [57], the emotional stressors associated with the effects of hospitalization in acute psychiatric care can be further understood [59, 60] and interventions for their management can be developed and implemented. However, further research is needed to investigate the specificity of various domains of generic PROMs (e.g. quality of life, recovery-oriented component of care, patient empowerment) [61] and dimensions of subjective well-being (e.g. satisfaction in life, sense of fulfillment, hope, optimism, connection), to better understand their distinctive contribution to the patient’s global perception of the quality of hospital care [57].

Future studies should also consider how to better contextualize the strength of the correlations between various domains of PROMs and assess the factors that might contribute to the discrepancies observed between PREMs and PROMs across diagnoses. They should also investigate if discrepancies in outcomes could be due to biases related to patients’ differences in sociodemographic background and clinical characteristics (such as age, family situation, education, severity of illness, comorbidities) or the type of care received (voluntary or involuntary treatment). Of particular interest would be research on specific clinical populations or profiles, such as patients with eating disorders, who report lower levels of outcome improvement and satisfaction with hospital care. It may be suggested that, more than in other groups of patients, the subjective well-being of patients with eating disorders depends on factors other than symptom improvement, for example, interfering personality traits such as “perfectionism” [62]. Exploring this hypothesis could shed valuable light on how to adapt clinical care strategies and interventions accordingly for this subset of patients.

Three limitations should be considered in the present study. Firstly, all data were collected in a single center, which may have resulted in selection bias as results might reveal the perception of patients who shared a similar experience of care. Nevertheless, patients were enrolled in different units treating different populations and using specific therapeutic approaches, delivered by separate care teams. In addition, patients reported significant differences in their perception of the quality of care. The study sample is also similar to other publications exploring relations between patient-reported measures of inpatients in psychiatric settings [33], and in other medical specialties [55, 63, 64]. Secondly, the most severe patients in an acute phase of the disease more often declined to be included in the study or failed to complete the assessments, which may result in possible attrition bias. However, considering the acuity of the timing of the data collection (within the first 48 hours of hospital admission), an attrition rate of 20.2% is relatively normative that is to say still in an acute phase. Lastly, this study focused on the appointed psychiatrists’ view of the caregiving experience, future studies could include other health professionals (e.g., nurses and psychologists) who spend significant time with service users in inpatient settings.

Despite the articulated limitations, this is one of the first studies to provide real data on the relationship between various domains of patient-reported measures from a large sample of patients receiving acute care in a French psychiatric hospital. It offers a unique insight into the value of specifically assessing subjective well-being with PROMs. These shed light on key aspects of care from the patient’s perspective that might otherwise go unassessed, and hence, unaddressed. As shown, the activity of implementing measures of subjective well-being may influence changes in clinical focus and improve the quality of care. For example, from the beginning of the hospital stay, by improving providers’ clinical judgment, identifying what is meaningful to patients, encouraging personalized treatment goals, and shared decision making. In addition, regularly measuring the patient’s well-being may provide valuable information on the evolution of these indicators in patients with chronic disorders that require long-term monitoring. Finally, the use of standardized indicators, such as those based on the OECD work on mental health, provides opportunities for data sharing, benchmarking, and sharing of best practices across settings.

In conclusion, this study strengthens existing research that standardized PROMs and PREMs can serve as key indicators of high-quality care, and supports the case for using PROMs of subjective well-being in clinical practice as relevant indicators in their own right for patients undergoing psychiatric hospital treatment.

## Data Availability

The datasets analyzed during the current study and that support its findings are available from the corresponding author on reasonable request.

## A. Appendix 1: Patient-reported outcome and experience measures questionnaires used for the study

*(nb: the participants completed the French version of the present questionnaire)*

**Table:**

***Patient-reported outcome measures (PROMs)*** I. World health organization well-being index (WHO-5)		
I have felt cheerful and in good spirits	All of the time; Most of the time; More than half of the time; Less than half of the time; Some of the time; At no time
I have felt calm and relaxed	All of the time; Most of the time; More than half of the time; Less than half of the time; Some of the time; At no time
I have felt active and vigorous	All of the time; Most of the time; More than half of the time; Less than half of the time; Some of the time; At no time
I woke up feeling fresh and rested	All of the time; Most of the time; More than half of the time; Less than half of the time; Some of the time; At no time
My daily life has been filled with things that interest me	All of the time; Most of the time; More than half of the time; Less than half of the time; Some of the time; At no time
*II. OECD Assessment of Subjective Well-being Items (core questions)*	
Overall, how satisfied are you with life as a whole these days?	[1–10] The question asks how satisfied you feel, on a scale from 0 to 10. Zero means you feel “not at all satisfied” and 10 means you feel “completely satisfied”	
Overall, to what extent do you feel the things you do in your life are worthwhile?	[1–10] The question asks how worthwhile you feel the things you do in your life are, on a scale from 0 to 10. Zero means you feel the things you do in your life are “not at all worthwhile,” and 10 “completely worthwhile”

**Table:**

***Patient-reported experience measures (PREMs)*** PREMs for Mental Health Care, adapted from OECD Set of Questions on Patient experiences with Ambulatory Care	
During the course of your treatment: Did your care providers treat you with courtesy and respect?	Yes, definitely; Yes, to some extent; No, not really; No, definitely not; Not sure; Decline to answer
Did your care providers spend enough time with you?	Yes, definitely; Yes, to some extent; No, not really; No, definitely not; Not sure; Decline to answer
Did your care providers explain things in a way that was easy to understand?	Yes, definitely; Yes, to some extent; No, not really; No, definitely not; Not sure; Decline to answer
Did your care providers involve you as much as you wanted to be in decisions about your care and treatment?	Yes, definitely; Yes, to some extent; No, not really; No, definitely not; Not sure; Decline to answer

## B. Appendix 2: Clinician-reported experience of care questionnaire used for the study

*(nb: the clinicians completed the French version of the present questionnaire)*

**Table:**

During the course of the treatment of your patient: Did you treat your patient with courtesy and respect?	Yes, definitely; Yes, to some extent; No, not really; No, definitely not; Not sure; Decline to answer
Did you spend enough time with your patient?	Yes, definitely; Yes, to some extent; No, not really; No, definitely not; Not sure; Decline to answer
Did you explain things to your patient in a way that was easy to understand?	Yes, definitely; Yes, to some extent; No, not really; No, definitely not; Not sure; Decline to answer
Did you involve your patient as much as he/she wanted to be in decisions about his/her care and treatment?	Yes, definitely; Yes, to some extent; No, not really; No, definitely not; Not sure; Decline to answer
